# Rhesus blood group haplotype determination by nanopore sequencing and adaptive sampling enables the precise determination of complex allele combinations that could not be accurately determined by standard methods

**DOI:** 10.1111/trf.70182

**Published:** 2026-04-08

**Authors:** Rebekka Waldmann, Marita Führer, Annika Vogt, Dominik Nolde, Melanie Riecker, Lennart Finn Herrlich, Timo Dinse, Hubert Schrezenmeier, Christof Weinstock, David Alexander Christian Messerer

**Affiliations:** ^1^ Institute for Clinical Transfusion Medicine and Immunogenetics Ulm German Red Cross Blood Service Baden‐Württemberg – Hessen and Ulm University Hospital Ulm Germany; ^2^ Institute for Transfusion Medicine Ulm University Hospital Ulm Germany

## Abstract

**Background:**

Patients with chronic transfusion needs such as those with sickle cell disease face a high risk of developing antibodies against high‐prevalence antigens in the RH blood group system, complicating transfusion therapy and potentially necessitating stem cell transplantation. Molecular characterization of the RH system is hindered by hybrid alleles and high sequence homology between *RHD* and *RHCE*, limiting the effectiveness of conventional short‐read sequencing.

**Study Design and Methods:**

We analyzed 11 control and 20 patient samples, some of which could not be reliably genotyped by standard methods.

**Results:**

Nanopore sequencing with adaptive sampling enables targeted, amplification‐free long‐read sequencing of the *RH* locus, resolving homologous and complex hybrid structures and enabling complete haplotype phasing for all samples, including samples that could not be accurately determined by standard methods like serology and short‐read sequencing. Four new alleles were identified and for 13 out of 20 patients the results led to a change in the transfusion regimen.

**Discussion:**

These findings show that nanopore sequencing with adaptive sampling allows unambiguous genotyping of the RH system, improves detection of complex variants, and supports better‐matched transfusion strategies for chronically transfused patients.

**Abbreviations**
BP: breakpoints
DNA: deoxyribonucleic acid
ISBT: International Society of Blood Transfusion
kb: kilobase
NGS: next‐generation sequencing
PCR: polymerase chain reaction
SCD: sickle cell disease
SCT: stem cell transplantation
SNP: single nucleotide polymorphism
SNVs: single nucleotide variants

## 1 INTRODUCTION

Patients with chronic transfusion needs for example those with sickle cell disease (SCD), have an elevated risk of red cell alloimmunization.^1^ Over 25% of patients with SCD of African descent carry variant *RHD* or *RHCE* alleles,^2^, ^3^ resulting in partial D or CE antigens that often lack high‐prevalence epitopes. When antibodies against high‐prevalence RH antigens are present, serological antigen and corresponding antibody analysis is technically complex and usually limited to specialized laboratories. These antibodies potentially make the provision of compatible red blood cell units extremely difficult or may contribute to the indication for stem cell transplantation (SCT).^4^ Accurate matching of RH phenotypes between patients and donors can help prevent alloimmunization, highlighting the importance of molecular methods in SCD transfusion management.^5^

The *RH* locus consists of two highly homologous genes, *RHD* and *RHCE*, with a gene sequence identity of 93.8%.^6^ In addition, these genes are prone to structural rearrangements such as deletions, duplications, and hybrid formations.^7^ Consequently, variants detected by single‐specific‐primer‐PCR or Sanger sequencing cannot be reliably assigned to a specific allele without extensive additional analyses, such as family member or complementary DNA studies. Moreover, primer drop out may lead to undetected clinically relevant variants.^8^

Short‐read next‐generation sequencing (NGS) faces similar limitations due to the high sequence similarity between RHD and RHCE and limited phasing capacity.^8^ For example, the long genetic distances between single nucleotide variants (SNVs) limit the ability of short‐read sequencing to resolve RH haplotypes. In contrast, long‐read nanopore sequencing overcomes these challenges. Previous studies have demonstrated the value of nanopore sequencing in analyzing *RHD* and other blood group genes.^9^, ^10^, ^11^ Until recently, these applications still required long‐range PCR for locus enrichment.

Here, we present a strategy for resolving complex RH haplotypes using adaptive sampling which has also been used for the analysis of multiple other blood groups in parallel by Gueuning et al.^12^ This amplification‐free approach avoids PCR‐related biases and laborious enrichment steps, provides sufficient coverage for both SNV and structural variant detection.^13^, ^14^ Most importantly, it enables accurate haplotype phasing across the entire *RH* locus, allowing for unambiguous genotyping.

## 2 MATERIAL AND METHODS

### 2.1 Patients and samples

Ethylenediaminetetraacetic acid blood or genomic DNA from 20 patients, for whom reliable determination of RH haplotypes was not possible, and blood from 11 healthy donors as control material were included in the study (obtained for diagnostic purposes by the Institute for Clinical Transfusion Medicine and Immunogenetics Ulm Single specific primer‐PCR reference laboratory). Further details are provided in the Supplemental Methods.

### 2.2 Sequencing and analysis

Genomic DNA was extracted and processed as described in the Supplemental Methods for library preparation and sequencing on the PromethION and MinION platforms (Oxford Nanopore Technologies). Adaptive sampling, a feature currently unique to nanopore sequencing, was applied to selectively enrich a 188 kb region on chromosome 1 encompassing the *RHD* and *RHCE* genes. Adaptive sampling is a software‐driven method for real‐time enrichment (or depletion) of specific DNA regions during sequencing, achieved by ejecting unwanted molecules from the nanopore while keeping target sequences. Real‐time, high‐accuracy basecalling was performed using MinKNOW. Downstream analysis included re‐basecalling, read filtering, structural and nucleotide variant detection, phasing, and haplotype assignment using custom R scripts, EPI2ME, and CLC Genomics Workbench. Final variant calls and haplotype assignments were manually validated and aligned with official International Society of Blood Transfusion (ISBT) alleles (detailed methods and workflow in Supplemental Methods and Figure S1).

## 3 RESULTS

### 3.1 Sequencing coverage and read lengths are sufficient for analysis

By combining nanopore sequencing and adaptive sampling, time‐ and cost‐efficient enrichment of the target region (chr1:25,252,509–25,440,825, ~188 kbp) was achieved (mean coverage of 65.6×, minimal mean coverage 30.4×, maximal mean coverage 113.7×) without the need for prior amplification (Supplemental Table 1).

Compared to control samples obtained from fresh blood, most patient samples contained DNA of low quality and quantity. To mitigate this, short fragments were removed by pulsed‐field electrophoresis, and only reads ≥2 kb were included in downstream bioinformatic analyses (see Figure S1). A median N50 of 8573 (min. 6402; max. 24,100) was achieved for all 31 samples (detailed sequence metrics in Table S1).

### 3.2 Phasing was possible for the entire *RH* locus

**FIGURE 1:**
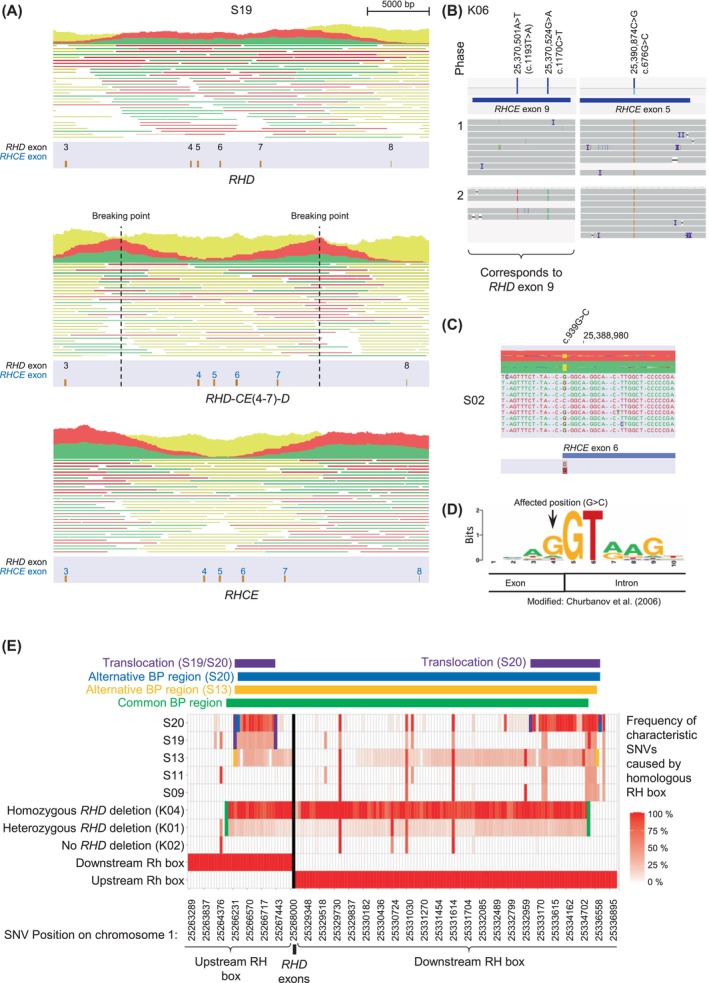
Examples of unexpected and/or challenging individual sequencing results. (A) Second alignment of sample S19 against an artificial reference sequence containing *RHD*, *RHD‐CE(4–7)‐D*, and *RHCE* to enable the assignment of the detected variants to the correct gene locus. Forward reads (green), reverse reads (red) and reads that have multiple, equally good alignments to the reference genome at different positions (yellow). (B) Representative reads of a regular RHCE*03 allele and a new heterozygous RHCE*03 allele in combination with a translocated *RHD* exon 9 of sample K06 underlying an RhCE phenotype of ccEE. (C) Detected nucleotide change next to the splice donor site of *RHCE* exon 6 in sample S02 and (D) conservation of nucleotides at splice donor sites according to Churbanov et al.^17^ (E) Analysis of Rhesus box recombination revealed a heterozygous deletion of *RHD* with alternative breakpoints (BP) for two samples (S13 and S20), the translocation of a downstream Rhesus box region into the upstream locus (samples S19 and S20) and the translocation of an upstream Rhesus box region into the downstream locus (S20). Nucleotide positions specific for the upstream or the downstream Rhesus box are depicted as boxes, the frequency of the nucleotide of the homologous Rhesus box at each position is represented by the color. Only a selection of the relevant positions is shown. All analyzed positions are shown in Figure S3. SNV, single nucleotide variant.

Successful phasing of all exon and splice‐site variants across all patient and control samples enabled clear haplotype determination (Table 1). While automated variant calling and haplotype assignment via the EPI2ME workflow was successful in most cases, samples with low‐quality alignments due to complex structural variations (e.g., exon translocations and deletions) required manual inspection and correction. For example, the common RHCE*02 (RHCE*Ce) allele, characterized by the translocation of *RHD* exon 2 into *RHCE*, consistently led to misaligned reads at the *RHD* exon 2 locus (Figure S2), impairing automated variant detection. Moreover, in samples S11 and S19 a large translocation of *RHCE* exons 4–7 into *RHD* did not allow for standard mapping and for automated variant calling. However, long reads spanning the breakpoints (BP) allowed precise identification of the translocation. This breakpoint information was used for manual realignment against an artificial reference (Figure 1A), enabling accurate variant calling and haplotype assignment.

**TABLE 1:** Determined RH haplotypes and observed obstacles regarding a fully automated evaluation. Novel alleles that cannot be assigned according to the official International Society of Blood Transfusion (ISBT) database are indicated in red. Green tick: serological or clinical information was confirmed by the sequencing derived phenotype; warning icon: sequencing derived phenotype specifies and/or extends the serological/clinical information and affects patients' future supply with blood.

	Serological or clinical information	ISBT alleles (nanopore sequencing)	Antigens coded by the alleles	Derived phenotype	Impact of the sequencing derived phenotype	Automated analysis and annotation was correct (yes/no)	Exon translocation detected
K01	D+ C+ E− c+ e+	RHD*01	D	D+ C+ E− c + e+	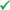	No: annotation failed	*RHCE‐D(2)‐CE*
		RHD*01N.01					
		RHCE*01	c, e				
		RHCE*02	C, e				
K02	D+ C+ E− c− e+	RHD*01 RHD*01 RHCE*02 RHCE*02	D D C, e C, e	D+ C+ E− c− e+	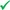	No: annotation failed	*RHCE‐D(2)‐CE*
K03	D+ C+ E− c+ e+	RHD*01	D	D+ C+ E− c+ e+	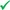	No: 4 SNVs *RHCE* Ex2 and 1 SNV in the splicing region of *RHCE* Ex2 were not detected, annotation failed, falsely called SNV (poly‐T‐stretch)	*RHCE‐D(2)‐CE*
		RHD*01N.01					
		RHCE*01	c, e				
		RHCE*02	C, e				
K04	D− C− E− c+ e+	RHD*01N.01		D− C− E− c + e+	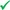	Yes	No
		RHD*01N.01					
		RHCE*01	c, e				
		RHCE*01	c, e				
K05	D+ C+ E−c + e+	RHD*01	D	D+ C+ E− c+ e+	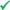	No: annotation failed, falsely called SNV (poly‐T‐stretch)	*RHCE‐D(2)‐CE*
		RHD*01N.01					
		RHCE*01	c, e				
		RHCE*02	C, e				
K06	D+ C− E+ c+ e−	RHD*01 RHD*01 RHCE*03 RHCE*03 variant *RHCE‐D(9)‐CE*	D D c, E plus a not yet defined cE antigen	D+ E+ c+ plus a not yet defined cE antigen	unknown	Yes	*RHCE‐D(9)‐CE*
K07	D+ C+ E− c+ e+	RHD*01	D	D+ C+ E− c+ e+	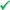	No: annotation failed	*RHCE‐D(2)‐CE*
		RHD*01N.01					
		RHCE*01	c, e				
		RHCE*02	C, e				
K08	D+ C− E+ c+ e+	RHD*01	D	D+ C− E+ c+ e+	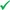	Yes	*No*
		RHD*01N.01					
		RHCE*01	c, e				
		RHCE*03	c, E				
K09	D+ C+ E+ c+ e+	RHD*01 RHD*01 RHCE*02 RHCE*03	D D C, e c, E	D+ C+ E+ c+ e+	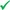	No: annotation failed	*RHCE‐D(2)‐CE*
K10	D+ C+ E+ c+ e+	RHD*01 RHD*01 RHCE*02 RHCE*03	D D C, e c, E	D+ C+ E+ c+ e+	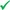	No: annotation failed	*RHCE‐D(2)‐CE*
K11	D− C− E− c+ e+	RHD*01N.01		D− C− E− c+ e+	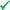	Yes	*No*
		RHD*01N.01					
		RHCE*01	c, e				
		RHCE*01	c, e				
S01	D+ C− E+ weak c + e+	RHD*01 RHD*01 RHCE*01.01 RHCE*03.01	D D c, e c, Ew, E partial	D+ C− E+ partial, weak c + e + weak Ew + (RH:11)	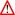	No: annotation failed	*No*
S02	D+ C+ weak E− c+ e+	RHD*01	D	D+ C+ weak E− c+ e+	Unknown	No: 5 SNV *RHCE* Ex2 and 1 SNV in the splicing region of *RHCE* Ex2 were not detected, annotation failed	*(No)* ^c^
		RHD*01N.01	c, e				
		RHCE*01					
		RHCE*02	C, e				
		*splicing might be affected*					
S03	D+ C+ E+ c+ e+ weak	RHD*01 RHD*01 RHCE*03 RHCE*02.22	D D c, E C, e partial	D+ C+ weak E+ c+ e + partial, weak	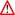	No: 1 SNV *RHCE* Ex2 was not detected, annotation failed	*RHCE‐D(2)‐CE*
S04	external laboratory requested sequence based typing of *RHD*	RHD*01 *new RHD allele c.802‐41_802‐38del* ^a^, RHCE*02 RHCE*02	D few or no D antigens C, e C, e	D+ C+ E− c− e+	unknown	No: annotation failed	*RHCE‐D(2)‐CE*
S05	D+ C+ weak E+ c+ e+ weak	RHD*01	D	D+ C+ weak E+ c+ e+ partial, weak	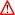	No: annotation failed	*RHCE‐D(2)‐CE*
		RHD*01N.01					
		RHCE*03	c, E				
		RHCE*02.22	C, e partial				
S06	D+ C+ weak E− c+ e+ anti‐C, −C^w^, −E, anti‐N, anti‐S	RHD*01	D	D+ C+ weak E− c+ e+	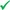	No: annotation failed	*RHCE‐D(2)‐CE* *RHCE‐D(5:667–5:787)‐CE*
		RHD*01N.01					
		RHCE*01	c, e				
		RHCE*02.04.01	C, e				
S07	D+ C− E+ c+ weak e+ weak	RHD*01 RHD*10.00 RHCE*03 RHCE*01.07.01	D D partial c, E c partial, e partial, hr^S^−, hr^B^−, CEVF−	D+ C− E+ c+ e+ partial, weak	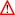	No: 1 SNV *RHCE* Ex1 was not detected, annotation failed	No
S08	D+ weak C+ weak E− c+ e+	RHD*01N.01 RHD*01 (43%) + *RHD variant (57%)* (mosaicism) RHCE*01 RHCE*02.08.01	D + theoretically a truncated D protein of 36 amino acids c, e C partial, e partial, C^w^, MAR−	D+ weak C+ partial E− c+ e+ C^W^+ (RH:8)	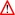 + poss. unknown effect	No: annotation failed	*RHCE‐D(2)‐CE*
S09	D+ C− E− c+ e+ weak transfusion with D− C− E− c+ e+ prior to admission suspected	RHD*01 RHD*01 RHCE*03 RHCE*01.20.01	D D c, E c partial, e partial V, VS, hr^B^ weak to neg	D+ C− E+ c+ e+ partial V+ (RH:10) VS+ (RH:20)	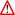	No: 2 SNVs *RHCE* Ex5 were not detected	No
S10	D+ C− E+ c+ e+ weak	RHD*01 RHD*10.00 RHCE*03 RHCE*01.07.01	D D partial c, E c partial, e partial hr^S^−, hr^B^−, CEVF−	D+ C− E+ c+ e+ partial, weak	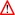	No: annotation failed	No
S11	D+ C+ weak E− c+ e+	RHD*01 *RHD‐CE(4–7)‐D* ^b^	D	D+ C− E− c+ e+ VS+ (RH:20)	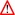	No: SNV calling in *RHD* Ex 4–7 was not possible, 1 SNV *RHCE* Ex5, 1 SNV *RHCE* Ex7 were not detected, annotation failed, falsely called SNV (poly‐T‐stretch)	*RHD‐CE(4–7)‐D*
		*+ c.733C>G* *+ c.1006G>T*					
		RHCE*01.01	c, e				
		RHCE*01.20.03	c partial, e partial VS, hr^B^−				
S12	D+ C+ weak E− c+ e+	RHD*01	D	D+ C+ weak E− c+ e+ LOCR+ (RH:55)	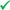	No: annotation failed	*RHCE‐D(2)‐CE*
		RHD*01N.01					
		RHCE*01	c, e				
		RHCE*02.11	C, e, LOCR				
S13	D+ C− E+ weak c+ e+	RHD*01	D	D+ C− E+ partial, weak c+ e+ partial, weak possibly CEAG− hr^B^−	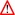	Yes	No
		RHD*01N.01					
		RHCE*03.04	c, E partial				
		RHCE *01.06.01	c, e partial CEAG−, hr^B^−				
S14	D− C+ E− c− e+	RHD*01N.01		D− C+ E− c− e+	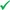	No: annotation failed	*RHCE‐D(2)‐CE*
		RHD*01N.01					
		RHCE*02	C, e				
		RHCE*02	C, e				
S15	D+ C+ weak E− c+ e+	RHD*01 RHD*10.00 RHCE*01.07.01 RHCE*02.10.01	D D partial c partial, e partial hr^S^−, hr^B^−, CEVF− C partial, e partial RH32, DAK, Sec−	D+ C+ partial, weak E− c+ partial, e+ partial, weak RH:32 DAK+ possibly hr^S^− hr^B^− CEVF− SEC−	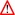	No: annotation failed	*RHCE‐D(2)‐CE* *RHCE‐D(4)‐CE*
S16	D+ C+ E+ c+ e+ weak	RHD*01 RHD*01 RHCE*02.22 RHCE*03.04	D D C, e partial c, E partial	D+ C+ weak E+ partial, weak c+ weak e+ partial, weak	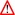	No: annotation failed	*RHCE‐D(2)‐CE*
S17	HDN unidentified antigen with low prevalence	RHD*01N.01		D− C− E− c+ e+	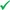	Yes	No
		RHD*01N.01					
		RHCE*01	c, e				
		RHCE*01	c, e				
S18	D+ C− E+ c+ e+ weak	RHD*01	D	D+ C− E+ c+ e+ weak Bea+ (RH:36)	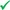	Yes	No
		RHD*01N.01					
		RHCE*03	c, E				
		RHCE*01.14	c, e, Bea				
S19	D+ C+ weak E− c+ e+	RHD*10.03 *RHD‐CE(4–7)‐D* ^b^ *+ c.733C>G*	D partial	D+ partial C− E− c+ e+ VS+ (RH:20)	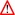	No: 1 SNV *RHCE* Ex5 was not detected, automated SNV calling in *RHD* Ex 4–7 was not possible, annotation failed	*RHD‐CE(4–7)‐D*
		*+ c.1006G>T*					
		RHCE*01.01	c, e				
		RHCE*01.20.03	c partial, e partial VS, hr^B^−				
S20	D+ C− E− c+ e+	RHD*01N.01		D+ partial C− E− c+ e+ V+ (RH:10) VS+ (RH:20)	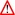	Yes	No
		RHD*09.03.01	D partial				
		RHCE*01	c, e				
		RHCE*01.20.01	c+ partial, e+ partial V, VS				

Abbreviations: Clair3, variant caller of the human variation workflow; SNPEff, annotation program of the human variation workflow; SNV, single nucleotide variant.

^a^ This allele closely resembles RHD*01EL.35 carrying c.802‐38_35del.

^b^ Allele designation according to RhesusBase^15^ and part of r'S Type 2 according to Reid et al.^16^ in combination with RHCE*01.20.03.

^c^ Automated SNV calling failed in the region of *RHCE* intron 1, exon 2 and intron 2 and a complete *RHD* exon 2 region translocation, as in the control samples, could not be confirmed/ruled out.

### 3.3 Sequencing‐based haplotypes match and confirm serological results

The serological results for all control samples matched the sequencing‐based haplotypes (Table 1). Interestingly, the ccEE phenotype of sample K06 was caused by one normal RHCE*03 allele and a second, previously unidentified RHCE*03 allele, carrying a translocated *RHD* exon 9 (*RHCE*‐*D(9)‐CE*) (Figure 1B).

### 3.4 Identification of new haplotypes, allowing clear resolution of previously ambiguous samples

Patient samples were successfully characterized, leading to the identification of three novel variant combinations that could not be assigned according to the official ISBT Blood Group Database^18^ (Table 1). One of those novel alleles involved an RHCE*02 variant with an additional mutation at the last nucleotide of exon 6 (*RHCE* c.939G>C, S02, Figure 1C) in a highly conserved^17^ (Figure 1D) splice donor site. A second novel allele was a standard RHD*01 with a 4‐nt deletion located 38 nt upstream of exon 6 (*RHD* c.802‐41_802‐38del, S04). This deletion is located outside the automatically analyzed regions for exons and splice sites (±20 nt) and was identified solely through manual review, due to its close resemblance to the RHD*01EL.35 (Del) allele with c.802‐37_34del.^18^

Finally, a third novel allele displayed a frameshift mutation (c.26_29dupTCCG), potentially resulting in a truncated Rh protein of 36 remaining amino acids (p.(Arg11ProfsTer26); sample S08). This variant was detected on one of the two alleles with a frequency of 57.14%, while the remaining reads corresponded to an RHD*01 allele (frequency 42.86%). The second allele of this sample was classified as RHD*01N.01. All detected sequence variants are listed in Table S2.

### 3.5 *RHD* deletion determination by Rhesus box analysis

*RHD* gene deletion results from homologous recombination between the upstream and downstream Rhesus boxes.^19^ Because this ~70 kbp structural variant was not detected in the EPI2ME workflow, we analyzed Rhesus box recombination by comparing characteristic nucleotide positions specific to each box by using the Rhesus box evaluation R‐script (Figures S3 and S4).

In all serologically D‐negative samples the previously described *RHD* deletion was confirmed^19^ (Figures 1E and S4). Additionally, 13 samples were heterozygous for this deletion.

In two samples with a heterozygous deletion of *RHD*, we detected alternative BP between position 25,266,231/25,266,266 and 25,336,558/25,336,593 (S13) and 25,266,266/25,266,499 and 25,336,593/25,336,826 of chromosome 1 (S20). Moreover, the Rhesus box analysis identified a translocation of a downstream Rhesus box segment into the upstream locus (S19, S20). Additionally, in S20, a translocated upstream Rhesus box segment was discovered in the downstream locus without *RHD* deletion.

## 4 DISCUSSION

Since *RHD* and *RHCE* genes are highly homologous^19^ and exon translocations are common,^20^ long reads are essential for accurate alignment and haplotype phasing. The nanopore sequencing with adaptive sampling, used in this study, enabled the precise determination of the RH haplotype for patient samples that could not be accurately determined by standard methods like serology and short‐read sequencing. The results of precise RH haplotype determination led to a change in transfusion support in 13 of 20 patients and may reduce the risk of alloimmunization in patients with chronic transfusion needs. This beneficial potential should be pursued further and investigated by future studies.

The ccEE phenotype of sample K06 illustrates that long‐read sequencing can uncover additional genetic complexity, even in serologically well‐characterized samples, and outperforms haplotype assignments derived from first‐ and second‐generation sequencing in combination with known SNP patterns.

The identification of the RHD*01 allele with a 4‐nt deletion located outside the routinely analyzed exon‐flanking regions in S04 and the additional mutation at the last nucleotide of exon 6 in S02 (a highly conserved splice donor site), which may likely affect the splicing at this position, highlights the need to include intronic regions in future analyses.

Furthermore, nanopore sequencing with adaptive sampling provides sufficient coverage to reliably detect variants, even those only present in a subpopulation of cells as shown for sample S08. Therefore, prior enrichment is unnecessary and amplification‐related biases such as primer‐related allele drop outs, potentially inadequate PCR products, and primer‐restricted definition of the sequencing regions can be avoided. In sample S08, a heterozygous *RHD* genotype with a mosaic pattern was identified. Of the reads assignable to one allele, 57% carried a novel *RHD* variant, where the remaining 43% corresponded to the RHD*01 type. In addition, a second allele, RHD*01N.01, was detected. This read distribution indicates a mosaicism, suggesting that approximately 57% of the cells carried an RHD*01N.01 allele in combination with a novel *RHD* variant, while the remaining 43% of cells carried the RHD*01N.01/RHD*01 allele combination. Mosaicisms have previously been described in the RH group system^21^, ^22^, ^23^ and may be of clinical relevance. However, it remains unclear whether this genetically detected mosaicism, identified in DNA derived from whole blood, is also present in nucleated precursors of the red blood cell lineage and may therefore lead to different Rhesus antigen characteristics.

**FIGURE 2:**
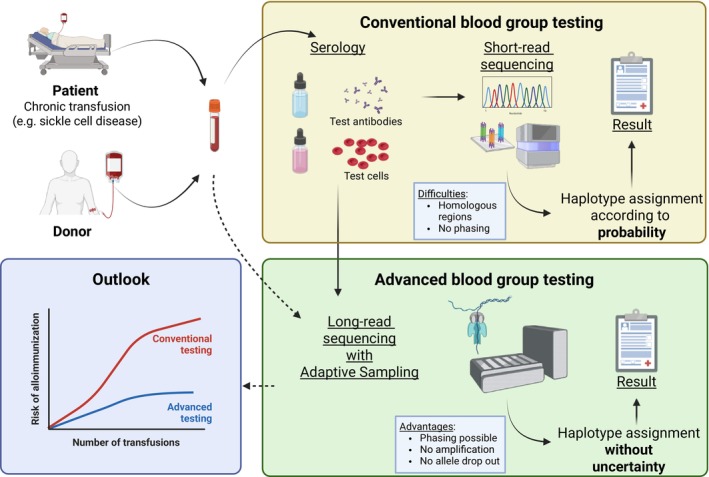
Comparison of conventional and advanced blood group testing in chronically transfused patients. Since conventional sequencing (first‐ and second‐generation sequencing) is problematic for large homologous regions, phasing of the entire RH locus is mostly impossible. A haplotype determination therefore relies only on the distribution of observed variants and their allocation to previously known haplotype patterns. Long‐read sequencing with adaptive sampling enables precise haplotype assignment, needs no previous amplification steps thereby avoiding allele drop outs, and will probably reduce alloimmunization risk in the future.

The complete sequencing and phasing of the Rhesus box region enabled the precise detection of *RHD* deletions and their distinction from Rhesus box translocations without *RHD* gene deletions, which is almost impossible by primer‐based methods. Taken together, these observations demonstrate the strength of long‐read sequencing in resolving complex *RH* genotypes and in detecting novel alleles, if high‐molecular weight DNA is available. Even complex hybrid structures were successfully resolved, demonstrating the robustness of the workflow, which can also be adapted to other blood group systems. As with all genetic methods, it should be noted that the phenotypic effects of new alleles and allele combinations can only be estimated and require further investigation. Although the available software for variant detection and annotation of nanopore sequencing data is insufficient for the automated analysis of the RH locus and needs further improvement for complete automation, this approach represents a major advancement toward precision medicine in transfusion care and will probably reduce alloimmunization risk in the future (Figure 2).

## CONFLICT OF INTEREST STATEMENT

All authors are employees of the German Red Cross Blood Service Baden‐Württemberg – Hessen.

## Supporting information


**Data S1.** Supporting Information.

## Data Availability

The data that support the findings of this study are available on request from the corresponding author. The data are not publicly available due to privacy or ethical restrictions.
