# Supplementary material for: Rhesus blood group haplotype determination by nanopore sequencing and adaptive sampling enables the precise determination of complex allele combinations that could not be accurately determined by standard methods
Source: Transfusion. 2026 Apr 8;66(6):1058–69. doi: 10.1111/trf.70182 (PMC13250356; doi:10.1111/trf.70182)
Supplement: Supplementary file 1 — Data S1. Supporting Information. [file TRF-66-1058-s001.pdf]

Brief report – Supplement

Title: Rhesus blood group haplotype determination by nanopore sequencing and adaptive sampling enables the precise determination of complex allele combinations that could not be accurately determined by standard methods

Short title: Rhesus Haplotype Sequencing by Adaptive Sampling

Rebekka Waldmann[1*], Marita Führer[1*], Annika Vogt[1], Dominik Nolde[1], Melanie Riecker[1], Lennart Finn Herrlich[2], Timo Dinse[1,2], Hubert Schrezenmeier[1,2], Christof Weinstock[1], David Alexander Christian Messerer[1,2,#]

[1]Institute for Clinical Transfusion Medicine and Immunogenetics Ulm, German Red Cross Blood Service Baden-Württemberg – Hessen and Ulm University Hospital, Ulm, Germany

[2]Institute for Transfusion Medicine, Ulm University Hospital, Ulm, Germany

* These authors contributed equally to this work.

[#]Correspondence:
David Alexander Christian Messerer
Institute for Clinical Transfusion Medicine and Immunogenetics Ulm
German Red Cross Blood Service Baden-Württemberg – Hessen and Ulm
University Hospital, Ulm, Germany
Email: d.messerer@blutspende.de; david.messerer@uni-ulm.de

Supplemental methods

Patients and samples

EDTA blood or genomic DNA from 20 patients, for whom reliable determination of RH haplotypes was not possible, were included in the study (obtained for diagnostic purposes by Institute for Clinical Transfusion Medicine and Immunogenetics Ulm Single specific primer-PCR reference laboratory). EDTA-anticoagulated blood from 11 healthy donors was used as control material, with prior informed consent obtained at the Institute for Clinical Transfusion Medicine and Immunogenetics Ulm (German Red Cross Blood Service Ulm). Serological RH typing had been performed previously and was confirmed using a second, independent sample. Ethical approval was obtained from the local ethics committee for samples from patients (approval number: #381/21). For anonymized residual samples from blood donors, a formal waiver from the local ethics committee of Ulm University was issued on 22 April 2024.

Sequencing

Genomic DNA was extracted from 1 ml EDTA blood using the Wizard Genomic DNA Purification Kit (Promega, #A1120, Madison, USA) according to the manufacturer's protocol. DNA was dissolved overnight at 4 °C in IDTE buffer (IDT, #11-05-01-05, San Diego, USA) and quantified using the Quant-iT dsDNA Assay Kit (Thermo Fisher Scientific, #Q33232, Eugene, USA). After DNA shearing with g-TUBEs (Covaris, #520079, Brighton, UK) at 6,000 rpm in an Eppendorf 5415 centrifuge for 1 min, DNA integrity was evaluated on agarose gels. If a high proportion of fragments <10 kbp was observed, size selection (>5 kbp) was performed after fragmentation using the BluePippin system (Sage Science) according to the manufacturer's instructions. 0.5–

3.0 µg DNA was used as input for library preparation using the DNA V14 kit (ONT, #SQK-LSK-114, Oxford, UK) and libraries were loaded onto PromethION R10.4.1 flow cells (FLO-PRO114M; all samples except K04) or a MinION R10.4.1 flow cell (FLO-MIN114; sample K04) and sequenced on a PromethION 24, PromethION 2 Integrated, or MinION Mk1B device for 10–72 hours. Flow cells were re-loaded, as needed, following a nuclease flush (ONT, EXP-WSH004) to increase sequencing yield. Adaptive Sampling was performed targeting a 188 kb region of the GRCh38 genome (chr1:25,252,509–25,440,825), using the Fast 400 bps model for real-time basecalling via MinKNOW with integrated Dorado. After sequencing, raw data was re-basecalled using the super accuracy model of MinKNOW (v7.4.12 or v7.4.14, ONT, Oxford, UK).

Analysis

Downstream analysis included re-basecalling, read filtering, structural and nucleotide variant detection, phasing, and haplotype assignment using custom R scripts, EPI2ME, and CLC Genomics Workbench. Final variant calls and haplotype assignments were manually validated and aligned with official ISBT alleles (detailed workflow in Supplemental Figure 1).

All R scripts used in this study are provided upon reasonable request to the corresponding author and were available during the review process. The script BamWidthFilter was applied to remove reads < 2,000 bp in length. Filtered, unaligned BAM files were analyzed with the EPI2ME Human Variation Workflow (configurations are listed below) to detect structural and nucleotide variants and to detect structural and nucleotide variants and to generate phased CRAM files aligned to the GRCh38 reference genome. Two positions (chr1:25,317,062 T>C and chr1:25,420,739 G>C)

were modified to match ISBT reference sequences[1] for *RHD* (NG_007494.1) and *RHCE* (NG_009208.3).

A VCF-Filter script was used to retain variants located in exons or splice sites (±20 nt). Haplotagged CRAM files were re-analyzed using the “Basic Variant Detection” tool in CLC Genomics Workbench (QIAGEN; configurations are listed below) with a 1% variant frequency threshold for RH box evaluation. The Rhesus Box Evaluation script was then used to identify upstream or downstream RH boxes by comparing characteristic nucleotide positions (see Supplemental Figure 1).

All exons and splice-site regions, as well as *RHD* deletions, were manually reviewed using Integrative Genomics Viewer (v2.18.4)[2]. All annotated variants and phasing results were manually verified by visual inspection of the individual aligned reads. SNVs in automatically misaligned reads were manually corrected and reassigned to the correct exon if automated variant calling failed due to complex structural variations (e.g., exon translocations and deletions). Haplotypes were assigned to official ISBT alleles based on the ISBT Blood Group Database[1].

EPI2ME human variation workflow configurations:

v2.4.1
https://github.com/epi2me-labs/wf-human-variation, ONT, Oxford, UK

sv	True	qdnaseq_bin_size	500
SNP	True	force_strand	False
cnv	False	depth_intervals	False
str	False	GVCF	False
mod	False	base_err	0.001
sample_name	gDNA164	gq_bin_size	5
bam_min_coverage	20	downsample_coverage	False
depth_window_size	25000	downsample_coverage_target	60
annotation	True	downsample_coverage_margin	1.1
phased	True	output_xam_fmt	cram
include_all_ctgs	False	threads	128
output_gene_summary	False	ubam_map_threads	8
output_report	True	ubam_sort_threads	3
haplocheck	False	ubam_bam2fq_threads	1
igv	False	modkit_threads	4
cluster_merge_pos	150	sv_benchmark_vcf	None
min_sv_length	30	sv_benchmark_bed	None
sv_benchmark	False	mitogenome	None
use_longphase	False	override_basecaller_cfg	None
ref_pct_full	0.1	clair3_model_path	None
var_pct_full	0.7	ctg_name	None
snp_min_af	0.08	vcf_fn	None
indel_min_af	0.15	tr_bed	None
min_cov	2	min_read_support	auto
min_mq	5	min_read_support_limit	2
min_qual	2	sniffles_args	None
min_contig_size	0	modkit_args	None
refine_snp_with_sv	True	sex	None
use_qdnaseq	False		

CLC Genomics configuration - basic variant detection:

v25.0.1

Ploidy = 2
Ignore positions with coverage above = 100.000
Restrict calling to target regions = Not set
Ignore broken pairs = No
Ignore non-specific matches = Reads
Minimum coverage = 2
Minimum count = 2
Minimum frequency (%) = 1,0

Base quality filter = No
Read direction filter = No
Relative read direction filter = No
Read position filter = No
Remove pyro-error variants = No
Create track = Yes
Create annotated table = Yes

Chromosomal regions/positions used for analysis as indicated:

Rhesus box characteristic SNV positions of NG_242261.1 (upstream RH box) and NG_242262.1 (downstream RH box, the A at position 7450 was deleted according to Wagner and Flegel)[3] used for RH box analysis.

chr	position	ref	var
1	25259092	C	
	TTGTTTTTTT		
1	25259103	C	T
1	25259180	G	A
1	25259197	G	A
1	25259218	G	A
1	25259230	A	-
1	25259339	T	C
1	25259363	G	A
1	25259368	T	C
1	25259389	G	A
1	25259412	T	A
1	25259572	A	G
1	25259577	G	A
1	25259579	C	T
1	25259605	TG	CA
1	25259638	G	A
1	25259650	A	G
1	25259660	G	A
1	25259686	A	T
1	25259832	C	T
1	25259889	C	T
1	25259997	T	C
1	25260001	C	T
1	25260031	T	C
1	25260165	T	C
1	25260185	C	T
1	25260195	G	A
1	25260256	C	T
1	25260285	C	T
1	25260323	A	G
1	25260348	-	TAA
1	25260386	G	A
1	25260441	C	T
1	25260570	T	C
1	25260583	G	T
1	25260620	A	T
1	25260696	C	T
1	25260769	C	T
1	25260864	C	G
1	25260927	G	A
1	25260951	G	A
1	25260960	T	C
1	25261022	C	G
1	25261104	T	C
1	25261154	G	A
1	25261234	T	G
1	25261250	A	C
1	25261280	C	T
1	25261288	A	G
1	25261330	T	C
1	25261350	C	G
1	25261365	C	G
1	25261401	TC	-
1	25261406	C	-
1	25261435	CA	TG
1	25261451	C	A

chr	position	ref	var
1	25261483	C	A
1	25261486	T	C
1	25261515	T	C
1	25261523	G	A
1	25261541	C	A
1	25261551	C	T
1	25261558	CCC	AGA
1	25261572	-	
	TTTTTTTTTTTTTTTTTTTTTTTTTGAG		
ACGGAGTCTCGCTCTGTTACCCAGGCTG			
GAGTGCAGTGGCGCGATCTCGGCTCACT			
GCAAGCTCCGCCTCCCGGGTTCACGCCA			
TTCTCCTGCCTCAGCCTCCGGAGTAGCTG			
GGACTACAGGCGCCCGCCACTACGCCCG			
GCTAAC			
1	25261746	G	C
1	25261756	G	A
1	25261795	TCTG	-
1	25261969	C	T
1	25262091	T	C
1	25262136	C	G
1	25262164	T	C
1	25262191	T	AAA
1	25262344	G	C
1	25262360	T	C
1	25262385	G	A
1	25262472	G	C
1	25262476	A	G
1	25262493	G	-
1	25262511	A	G
1	25262569	G	A
1	25262633	C	T
1	25262708	A	G
1	25262721	A	C
1	25262829	T	C
1	25262837	C	A
1	25262844	A	G
1	25262870	T	C
1	25263026	G	A
1	25263174	G	C
1	25263276	A	G
1	25263289	C	T
1	25263338	G	C
1	25263528	T	-
1	25263539	A	G
1	25263807	T	C
1	25263837	G	A
1	25263987	-	C
1	25264125	G	A
1	25264297	T	C
1	25264315	A	C
1	25264376	A	G
1	25264479	C	T
1	25264584	C	T
1	25266048	A	-
1	25266137	G	A
1	25266231	C	A

chr	position	ref	var
1	25266266	C	G
1	25266499	T	G
1	25266528	TA	-
1	25266560	ATC	GCA
1	25266570	-	AAAC
1	25266580	C	A
1	25266639	T	C
1	25266696	T	C
1	25266711	A	C
1	25266717	G	A
1	25266729	A	G
1	25266764	C	T
1	25266769	C	T
1	25266962	T	C
1	25267443	A	G
1	25267675	G	T
1	25267744	A	C
1	25267995	C	G
1	25267997	G	T
1	25268000	G	A
1	25269999	Boxen	Trennung
1	25329235	TTGTTTTTT	C
1	25329254	T	C
1	25329331	A	G
1	25329348	A	G
1	25329369	A	G
1	25329381	-	A
1	25329489	C	T
1	25329513	A	G
1	25329518	C	T
1	25329539	A	G
1	25329562	A	T
1	25329723	G	A
1	25329728	A	G
1	25329730	T	C
1	25329756	CA	TG
1	25329789	A	G
1	25329801	G	A
1	25329811	A	G
1	25329837	T	A
1	25329983	T	C
1	25330040	T	C
1	25330148	C	T
1	25330152	T	C
1	25330182	C	T
1	25330316	C	T
1	25330336	T	C
1	25330346	A	G
1	25330407	T	C
1	25330436	T	C
1	25330474	G	A
1	25330499	TAA	-
1	25330540	A	G
1	25330595	T	C
1	25330724	C	T
1	25330737	T	G
1	25330774	T	A
1	25330850	T	C
1	25330923	T	C
1	25331030	G	C
1	25331093	A	G
1	25331117	A	G
1	25331126	C	T

chr	position	ref	var
1	25331188	G	C
1	25331270	C	T
1	25331320	A	G
1	25331400	G	T
1	25331416	C	A
1	25331446	T	C
1	25331454	G	A
1	25331496	C	T
1	25331516	G	C
1	25331531	G	C
1	25331598	TG	CA
1	25331614	A	C
1	25331646	A	C
1	25331649	C	T
1	25331678	C	T
1	25331686	A	G
1	25331704	A	C
1	25331714	T	C
1	25331721	AGA	CCC
1	25331735	TTTTTTTTTTTTTTTTTTTTTTTTGAG<br>ACGGAGTCTCGCTCTGTTACCCAGGCTG<br>GAGTGCAGTGGCGCGATCTCGGCTCACT<br>GCAAGCTCCGCCTCCCGGGTTCACGCCA<br>TTCTCCTGCCTCAGCCTCCGGAGTAGCTG<br>GGACTACAGGCGCCCGCCACTACGCCCG<br>GCTAAC	-
1	25332075	C	G
1	25332085	A	G
1	25332124	-	TCTG
1	25332294	T	C
1	25332416	C	T
1	25332461	G	C
1	25332489	C	T
1	25332516	AAA	T
1	25332671	C	G
1	25332687	C	T
1	25332712	A	G
1	25332799	C	G
1	25332803	G	A
1	25332820	-	G
1	25332837	G	A
1	25332895	A	G
1	25332959	T	C
1	25333034	G	A
1	25333047	C	A
1	25333155	C	T
1	25333163	A	C
1	25333170	G	A
1	25333196	C	T
1	25333352	A	G
1	25333500	C	G
1	25333602	G	A
1	25333615	T	C
1	25333664	C	G
1	25333854	-	T
1	25333864	G	A
1	25334132	C	T
1	25334162	A	G
1	25334312	C	-
1	25334451	A	G
1	25334623	C	T
1	25334641	C	A

chr	position	ref	var	chr	position	ref	var
1	25334702	G	A	1	25337025	C	T
1	25334805	T	C	1	25337040	C	A
1	25334910	T	C	1	25337046	A	G
1	25336375	-	A	1	25337058	G	A
1	25336464	A	G	1	25337093	T	C
1	25336558	A	C	1	25337098	T	C
1	25336593	G	C	1	25337291	C	T
1	25336826	G	T	1	25337772	G	A
1	25336855	-	TA	1	25338004	T	G
1	25336885	GCA	ATC	1	25338073	C	A
1	25336895	AAAC	-	1	25338324	G	C
1	25336909	A	C	1	25338326	T	G
1	25336968	C	T	1	25338329	A	G

Characteristic variations of homopolymer regions were not taken into account.

Red marked SNVs define the end of the identical region and arise from the deletion of the A at position 7450 of NG_242262.1 (downstream RH box) as previously published[3]. This position varies from the hg38 reference sequence (insertion of A) but was not changed in the reference sequence used to avoid differences in the annotation of detected variations compared to standard hg38. This distinct position was manually evaluated for the RH box analysis and the frequency of SNVs at this position were manually added to the sample’s .csv-file.

Supplemental Tables

Supplemental Table 1: Sequencing metrics and individual variant-calling parameters. *raw data was saved in the .pod5 file format without pass/fail separation leading to a large proportion of included failed reads. For all other samples the .fast5 file format was used with pass/fail separation and only passed reads were used. Samples were excluded for the calculation of the mean value and min./max. determination.

#	DNA Size Selection via pulsed-field electrophoresis (high-pass filter)	Sequencing Device	Read Quality (Mean)	Accuracy (Mean)	Percentage of reads ≥ 2,000 bp [%]	Total Reads (≥ 2,000 bp)	N50 of reads ≥ 2,000 bp	Mean Coverage	Variants detected for phasing
K01	no	P2	18.3	98.0	93.7	1063	15154	60.0	364
K02	no	P24	19.6	98.4	8.2	2398	10479	108.7	217
K03	no	P2	18.8	98.2	93.2	1673	10706	75.7	229
K04	no	MinION	19.2	98.3	33.2	618	13818	30.4	247
K05	no	P24	17.0	97.4	89.1	1737	7057	55.6	377
K06	no	P2	18.3	98.1	91.6	1168	11436	54.1	316
K07	no	P24	18.8	98.2	94.3	1666	8063	63.1	402
K08	no	P2	17.4	97.7	0.2*	784	20553	55.3	224
K09	no	P2	19.1	98.4	0.2*	593	24100	39.9	339
K10	no	P24	19.1	98.3	93.4	1768	9004	74.6	288
K11	no	P2	18.0	98.0	87.1	1115	13535	54.7	267
S01	yes (≥7 kbp)	P2	17.5	97.8	95.7	1515	11004	83.8	220
S02	yes (≥5 kbp)	P24	17.7	97.8	96.4	2133	7251	78.4	251
S03	yes (≥7 kbp)	P2	16.4	97.2	97.6	1270	10976	71.5	321
S04	yes (≥5 kbp)	P24	18.9	98.3	91.8	2190	7286	75.5	230
S05	yes (≥5 kbp)	P24	19.6	98.4	95.3	1561	9328	69.9	340
S06	yes (≥5 kbp)	P24	17.9	98.0	94.2	1203	7851	46.5	348
S07	yes (≥5 kbp)	P2	16.9	97.5	96.0	2465	7544	89.6	257
S08	yes (≥5 kbp)	P24	17.7	97.9	95.5	1162	8812	49.9	330
S09	yes (≥7 kbp)	P2	16.7	97.4	96.1	2297	10430	113.7	219
S10	yes (≥5 kbp)	P24	16.2	97.2	95.7	2052	6402	68.5	276
S11	yes (≥5 kbp)	P24	17.7	97.9	95.0	2270	7436	83.4	285
S12	yes (≥5 kbp)	P24	18.6	98.2	92.9	1515	7500	54.7	312
S13	yes (≥7 kbp)	P2	19.4	98.4	94.3	1218	9905	57.1	340
S14	yes (≥5 kbp)	P24	17.3	97.5	95.8	1176	8565	48.4	322
S15	yes (≥5 kbp)	P24	19.7	98.5	92.4	1400	8315	56.4	340
S16	yes (≥5 kbp)	P24	17.0	97.5	93.1	1743	7430	64.4	314
S17	yes (≥5 kbp)	P24	16.3	97.2	92.7	1654	6684	48.1	268
S18	no	P2	17.4	97.8	95.9	2031	8573	77.8	227
S19	no	P24	20.3	98.5	97.2	2062	7897	77.3	389
S20	no	P24	17.1	97.5	94.1	1360	7509	46.8	448
		**Mean**	**18.1**	**97.9**	**89.0**	-	-	**65.6**	**300**
		**Median**	**-**	**-**	**-**	**1561**	**8573**	**63.1**	**-**
		**Min**	**16.2**	**97.2**	**8.2**	**593**	**6402**	**30.4**	**217**
		**Max**	**20.3**	**98.5**	**97.6**	**2465**	**24100**	**113.7**	**448**

Supplemental Table 2: Overview of all detected sequence variations and the corresponding ISBT allele assignment. Alleles with the indicated breakpoint are marked with x. The dash indicates that no sequence variation was found. Alternative breakpoints and variations that could not be assigned to official ISBT alleles according to the Blood Group Database[1] are marked in red. ‡ This allele closely resembles RHD*01EL.35 (Del) with c.802-38_35del, ‡‡ allele designation according to RhesusBase[4] and part of r'S Type 2 according to Reid et al.[5] in combination with *RHCE**01.20.03.

#	Gene	Rhesus box analysis: breakpoint detected?	BP (start region - end region)	Allele 1	Allele 2	ISBT allele 1	ISBT allele 2
K01	*RHD*	yes	25,264,584 / 25,266,048 - 25,334,910 / 25,336,375	x	-	*RHD**01N.01	*RHD**01
	RHCE			-	c.307C>T c.203A>G c.201A>G c.178C>A c.150C>T c.48G>C	*RHCE**01	*RHCE**02
K02	*RHD*	no	-	-	-	*RHD**01	*RHD**01
	RHCE			c.307C>T c.203A>G c.201A>G c.178C>A c.150C>T c.48G>C	c.307C>T c.203A>G c.201A>G c.178C>A c.150C>T c.48G>C	*RHCE**02	*RHCE**02
K03	*RHD*	yes	25,264,584 / 25,266,048 - 25,334,910 / 25,336,375	x	-	*RHD**01N.01	*RHD**01
	RHCE			-	c.307C>T c.203A>G c.201A>G c.178C>A c.150C>T c.48G>C	*RHCE**01	*RHCE**02
K04	*RHD*	yes	25,264,584 / 25,266,048 - 25,334,910 / 25,336,375	x	x	*RHD**01N.01	*RHD**01N.01
	RHCE			-	-	*RHCE**01	*RHCE**01
K05	*RHD*	yes	25,264,584 / 25,266,048 - 25,334,910 / 25,336,375	x	-	*RHD**01N.01	*RHD**01
	RHCE			-	c.307C>T c.203A>G c.201A>G c.178C>A c.150C>T c.48G>C	*RHCE**01	*RHCE**02
K06	*RHD*	no	-	-	-	*RHD**01	*RHD**01
	RHCE			c.676G>C	c.1193T>A c.1170C>T c.676G>C	*RHCE**03	new allele *RHCE**03 in combination with *RHCE-D(9)-CE*
K07	*RHD*	yes	25,264,584 / 25,266,048 - 25,334,910 / 25,336,375	x	-	*RHD**01N.01	*RHD**01
	RHCE			-	c.307C>T c.203A>G c.201A>G c.178C>A c.150C>T c.48G>C	*RHCE**01	*RHCE**02
K08	*RHD*	yes	25,264,584 / 25,266,048 - 25,334,910 / 25,336,375	-	x	*RHD**01	*RHD**01N.01
	RHCE			c.676G>C	-	*RHCE**03	*RHCE**01
K09	*RHD*	no	-	-	-	*RHD**01	*RHD**01
	RHCE			c.676G>C	c.307C>T c.203A>G c.201A>G c.178C>A c.150C>T c.48G>C	*RHCE**03	*RHCE**02
K10	*RHD*	no	-	-	-	*RHD**01	*RHD**01
	RHCE			c.676G>C	c.307C>T c.203A>G c.201A>G c.178C>A c.150C>T c.48G>C	*RHCE**03	*RHCE**02
K11	*RHD*	yes	25,264,584 / 25,266,048 - 25,334,910 / 25,336,375	x	x	*RHD**01N.01	*RHD**01N.01
	RHCE			-	-	*RHCE**01	*RHCE**01
S01	*RHD*	no	-	-	-	*RHD**01	*RHD**01
	RHCE			c.676G>C c.500T>A	c.48G>C	*RHCE**03.01	*RHCE**01.01
S02	*RHD*	yes	25,264,584 / 25,266,048 - 25,334,910 / 25,336,375	-	x	*RHD**01	*RHD**01N.01
	RHCE			c.939G>C (p.Pro313Pro) c.307C>T c.203A>G c.201A>G c.178C>A c.150C>T c.48G>C	-	*RHCE**02 possibly with affected splicing	*RHCE**01
S03	*RHD*	no	-	-	-	*RHD**01	*RHD**01
	RHCE			c.667G>T c.307C>T c.203A>G c.201A>G c.178C>A c.150C>T c.48G>C	c.676G>C	*RHCE**02.22	*RHCE**03
S04	*RHD*	no	-	-	c.802-46_802-43del (bzw. c.802-41_802-38del)	*RHD**01	new allele (*RHD* c.802-41_802-38del)‡
	RHCE			c.307C>T c.203A>G c.201A>G c.178C>A c.150C>T c.48G>C	c.307C>T c.203A>G c.201A>G c.178C>A c.150C>T c.48G>C	*RHCE**02	*RHCE**02
S05	*RHD*	yes	25,264,584 / 25,266,048 - 25,334,910 / 25,336,375	x	-	*RHD**01N.01	*RHD**01
	RHCE			c.676G>C	c.667G>T c.307C>T c.203A>G c.201A>G c.178C>A c.150C>T c.48G>C	*RHCE**03	*RHCE**02.22
S06	*RHD*	yes	25,264,584 / 25,266,048 - 25,334,910 / 25,336,375	x	-	*RHD**01N.01	*RHD**01
	RHCE			-	c.787A>G c.744T>C c.733C>G c.712A>G c.697C>G c.667G>T c.307C>T c.203A>G c.201A>G c.178C>A c.150C>T c.48G>C	*RHCE**01	*RHCE**02.04.01
S07	*RHD*	no	-	c.1136C>T	-	*RHD**10.00	*RHD**01
	RHCE			c.667G>T c.48G>C	c.676G>C	*RHCE**01.07.01	*RHCE**03

S08	RHD	yes	25,264,584 / 25,266,048 - 25,334,910 / 25,336,375	x	57,14 % c.26_29dupTCCG (p.(Arg11ProfsTer26)) (chimerism)	RHD*01N.01	truncated RHD protein of theoretically 36 AS + RHD*01
	RHCE			-	c.307C>T c.203A>G c.201A>G c.178C>A c.150C>T c.122A>G c.48G>C	RHCE*01	RHCE*02.08.01
S09	RHD	no	-	-	-	RHD*01	RHD*01
	RHCE			c.733C>G	c.676G>C	RHCE*01.20.01	RHCE*03
S10	RHD	no	-	c.1136C>T	-	RHD*10.00	RHD*01
	RHCE			c.667G>T c.48G>C	c.676G>C	RHCE*01.07.01	RHCE*03
S11	RHD	no	-	c.505A>C c.509T>G c.514A>T c.544T>A c.577G>A c.594A>T c.602C>G c.667T>G c.697G>C c.712G>A c.744C>T c.787G>A c.800A>T c.916G>A c.932A>G c.941G>T c.968C>A c.974G>T c.979A>G c.985G>C c.986G>A c.989A>C c.992A>T c.1025T>C c.1048G>C c.1053C>T c.1057G>T c.1059A>G c.1060G>A c.1061C>A c.1006G>T		RHD-CE(4-7)-D ‡‡ + c.733C>G + c.1006G>T	RHD*01
	RHCE			c.1006G>T c.733C>G c.48G>C	c.48G>C	RHCE*01.20.03	RHCE*01.01
S12	RHD	yes	25,264,584 / 25,266,048 - 25,334,910 / 25,336,375	x		RHD*01N.01	RHD*01
	RHCE			-	c.307C>T c.286G>A c.203A>G c.201A>G c.178C>A c.150C>T c.48G>C	RHCE*01	RHCE*02.11
S13	RHD	yes	25.266.231 / 25.266.266 - 25.336.558 / 25.336.593	-	x	RHD*01	RHD*01N.01
	RHCE			c.676G>C c.602G>C	c.254C>G	RHCE*03.04	RHCE01.06.01
S14	RHD	yes	25,264,584 / 25,266,048 - 25,334,910 / 25,336,375	x	x	RHD*01N.01	RHD*01N.01
	RHCE			c.307C>T c.203A>G c.201A>G c.178C>A c.150C>T c.48G>C	c.307C>T c.203A>G c.201A>G c.178C>A c.150C>T c.48G>C	RHCE*02	RHCE*02
S15	RHD	no	-	-	c.1136C>T	RHD*01	RHD*10.00
	RHCE			c.602G>C c.594T>A c.577A>G c.544A>T c.514T>A c.509G>T c.505C>A c.307C>T c.203A>G c.201A>G c.178C>A c.150C>T c.48G>C	c.667G>T c.48G>C	RHCE*02.10.01	RHCE*01.07.01
S16	RHD	no	-	-	-	RHD*01	RHD*01
	RHCE			c.676G>C c.602G>C	c.667G>T c.307C>T c.203A>G c.201A>G c.178C>A c.150C>T c.48G>C	RHCE*03.04	RHCE*02.22
S17	RHD	yes	25,264,584 / 25,266,048 - 25,334,910 / 25,336,375	x	x	RHD*01N.01	RHD*01N.01
	RHCE			-	-	RHCE*01	RHCE*01
S18	RHD	yes	25,264,584 / 25,266,048 - 25,334,910 / 25,336,375	x	-	RHD*01N.01	RHD*01
	RHCE			c.676G>C	c.662C>G	RHCE*03	RHCE*01.14
S19	RHD	no	-	c.505A>C c.509T>G c.514A>T c.544T>A c.577G>A c.594A>T c.602C>G c.667T>G c.697G>C c.712G>A c.744C>T c.787G>A c.800A>T c.916G>A c.932A>G c.941G>T c.968C>A c.974G>T c.979A>G c.985G>C c.986G>A c.989A>C c.992A>T c.1025T>C c.1048G>C c.1053C>T c.1057G>T c.1059A>G c.1060G>A c.1061C>A c.1006G>T	c.835G>A c.1136C>T	RHD-CE(4-7)-D ‡‡ + c.733C>G + c.1006G>T	RHD*10.03
	RHCE			c.1006G>T c.733C>G c.48G>C	c.48G>C	RHCE*01.20.03	RHCE*01.01
S20	RHD	yes	25.266.266 / 25.266.499 - 25.336.593 / 25.336.826	x	c.602C>G c.667T>G c.819G>A	RHD*01N.01	RHD*09.03.01
	RHCE			c.733C>G	-	RHCE*01.20.01	RHCE*01

Supplemental Figures

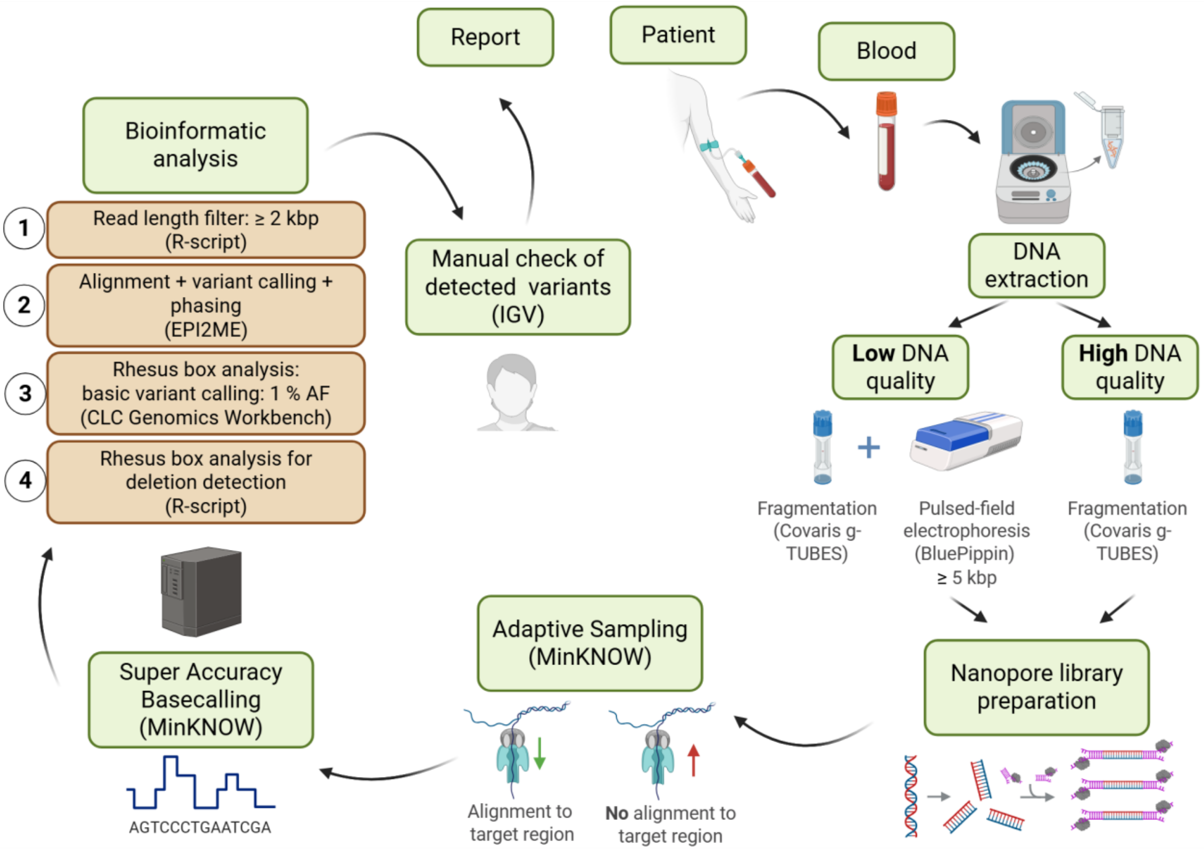

Supplemental Figure 1: Graphical summary of the workflow for *RHD*/*RHCE* haplotype analysis via third-generation sequencing with adaptive sampling. AF = allele frequency, IGV = Integrative Genomics Viewer.

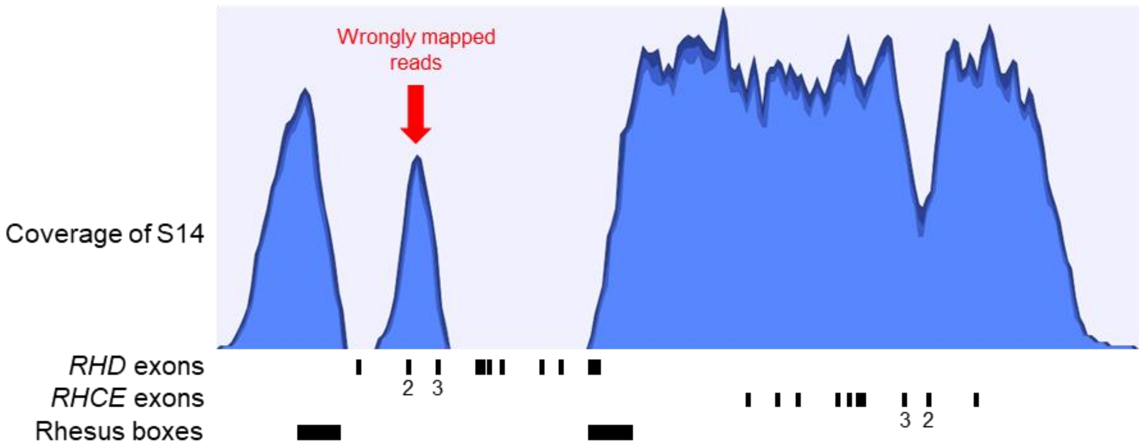

Supplemental Figure 2: Coverage of sample S14 with *RHD**01N.01 / *RHD**01N.01 (D-/D-) and *RHCE**02 / *RHCE**02 (Ce/Ce). *RHCE**02 is characterized by a translocation of *RHD* exon 2 into *RHCE* and leads to incorrectly mapped reads at the *RHD* exon 2 locus and a reduced coverage at *RHCE* exon 2.

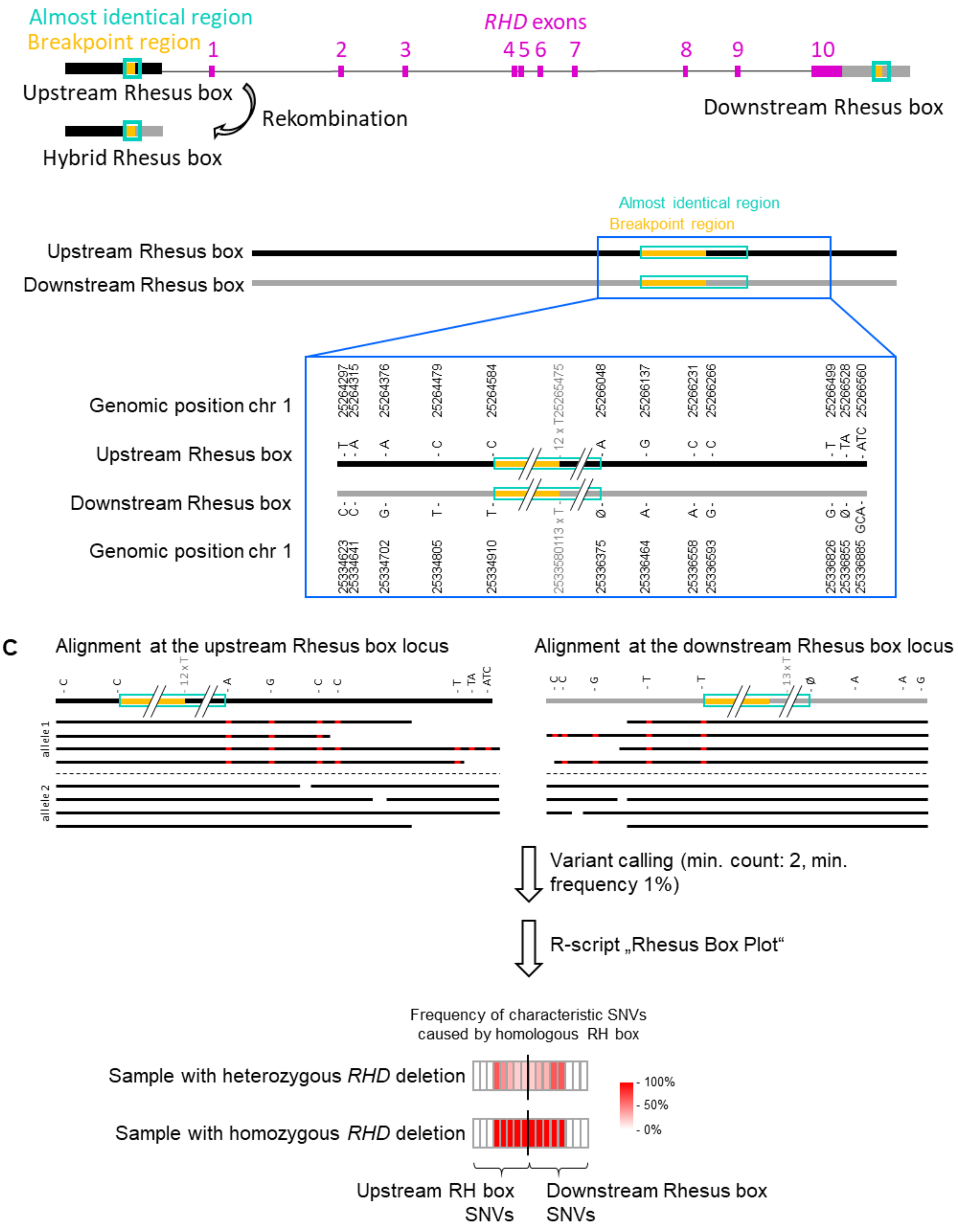

Supplemental Figure 3: Visual representation of the Rhesus box analysis. (A) Genomic locus of *RHD* with upstream Rhesus box, downstream Rhesus box and the breakpoint region in which recombination occurs, leading to the deletion of *RHD*. (B) Example of characteristic nucleotide positions that enable the identification of the upstream or downstream Rhesus box. (C) Workflow of the Rhesus box analysis.

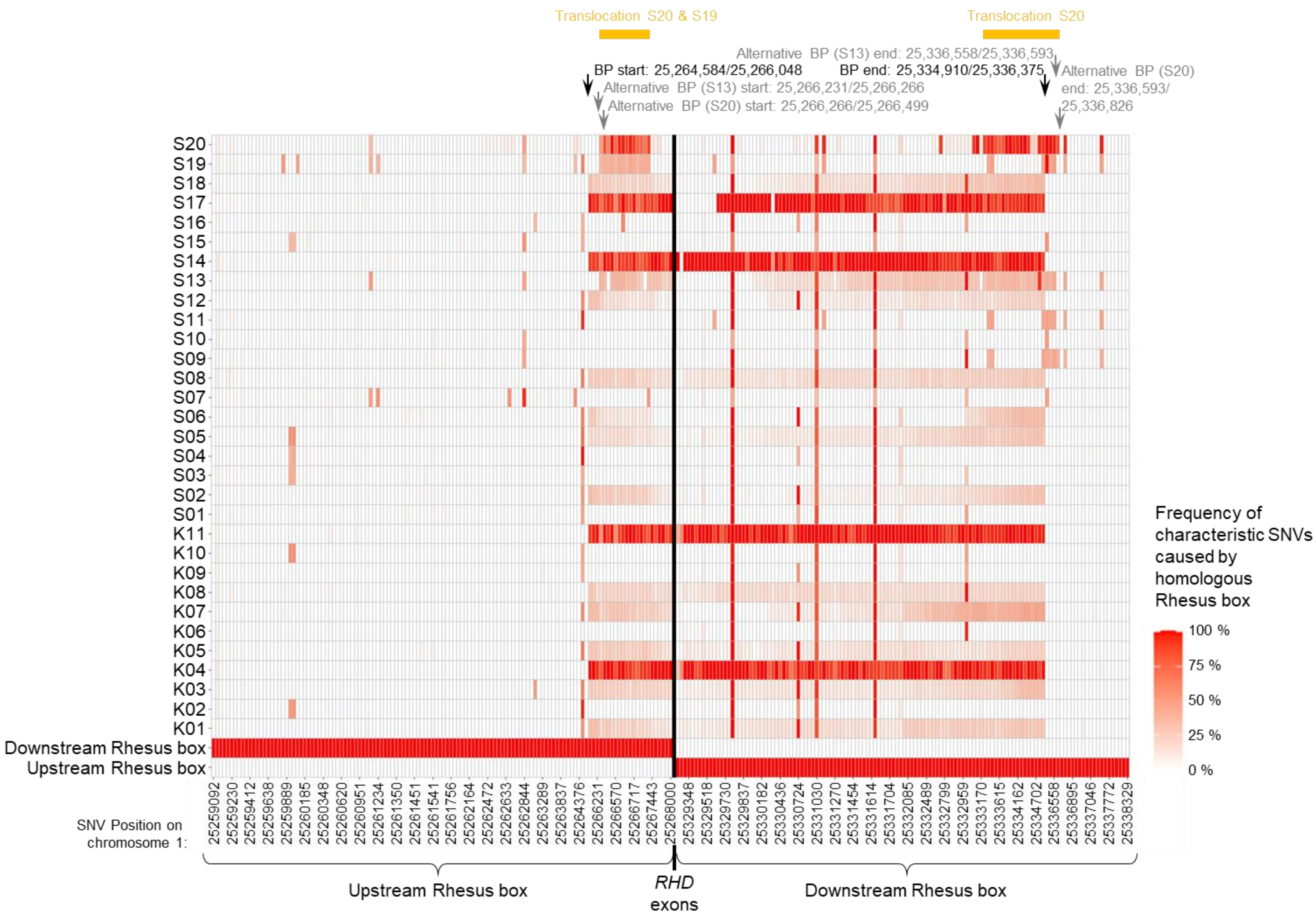

Supplemental Figure 4: Rhesus box analysis of all samples. A heterozygous deletion of *RHD* with alternative breakpoints (BP) was detected for samples S13 and S20. The translocation of a downstream Rhesus box region into the upstream locus was detected for sample S19 and S20 and the translocation of an upstream Rhesus box region into the downstream locus for S20 (yellow boxes). Single nucleotide variant (SNV) positions specific for the upstream or the downstream Rhesus box are depicted as boxes, the frequency of the nucleotide of the homologous Rhesus box at each position is represented by the color. All analyzed SNV positions are listed in the supplement.

References

1. International Society of Blood Transfusion. Blood Group Database. In. 2025. (Version: 137 Updated: 24 Mar 2025). Available from: https://www.blooddatabase.org/system/RH

2. Robinson JT, Thorvaldsdóttir H, Winckler W, Guttman M, Lander ES, Getz G, et al. Integrative Genomics Viewer. Nat Biotechnol. 2011 Jan;29(1):24–6. doi:10.1038/nbt.1754

3. Wagner FF, Flegel WA. RHD gene deletion occurred in the Rhesus box. Blood. 2000 Jun 15;95(12):3662–8. PubMed PMID: 10845894.

4. Wagner FF and Flegel WA. The Human RhesusBase [Internet]. 2014 [cited 2025 May 1]. Available from: https://rhesusbase.info/

5. Reid ME, Lomas-Francis C, Olsson ML. Rh Blood Group System. In: The Blood Group Antigen FactsBook [Internet]. Elsevier; 2012 [cited 2025 Jun 2]. p. 147–262. Available from: https://linkinghub.elsevier.com/retrieve/pii/B9780124158498000065 doi:10.1016/B978-0-12-415849-8.00006-5
